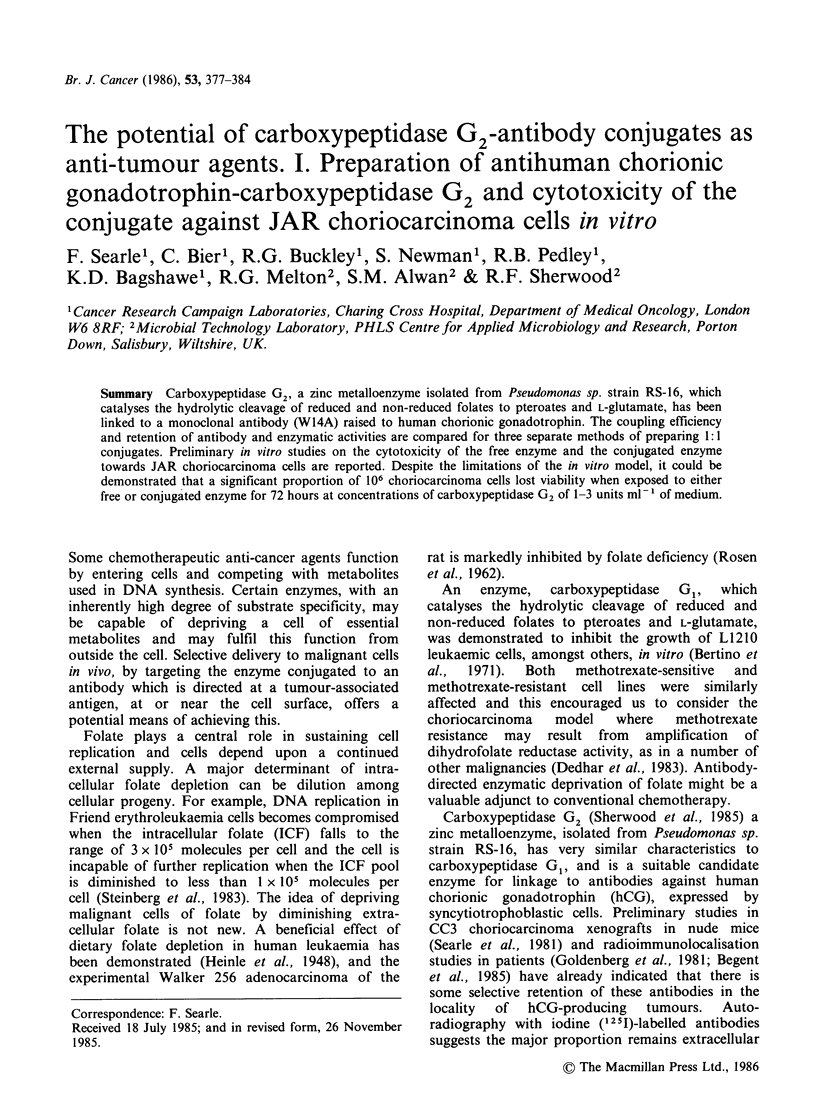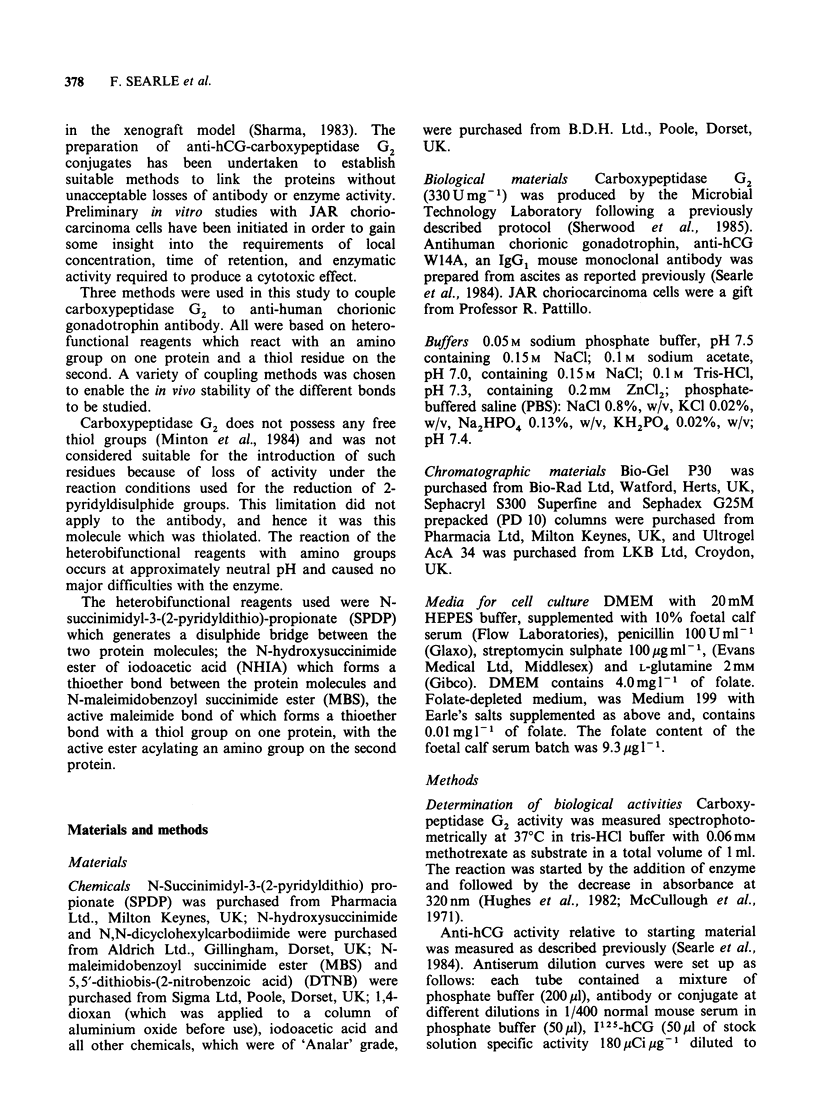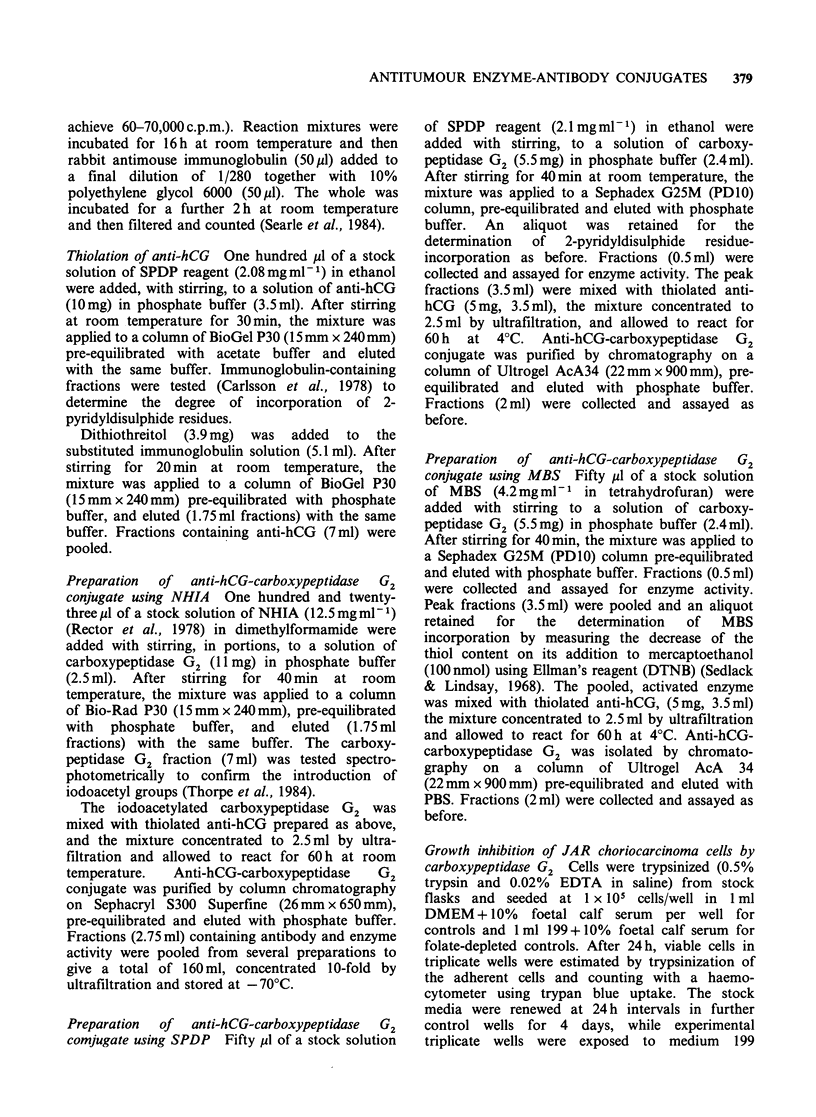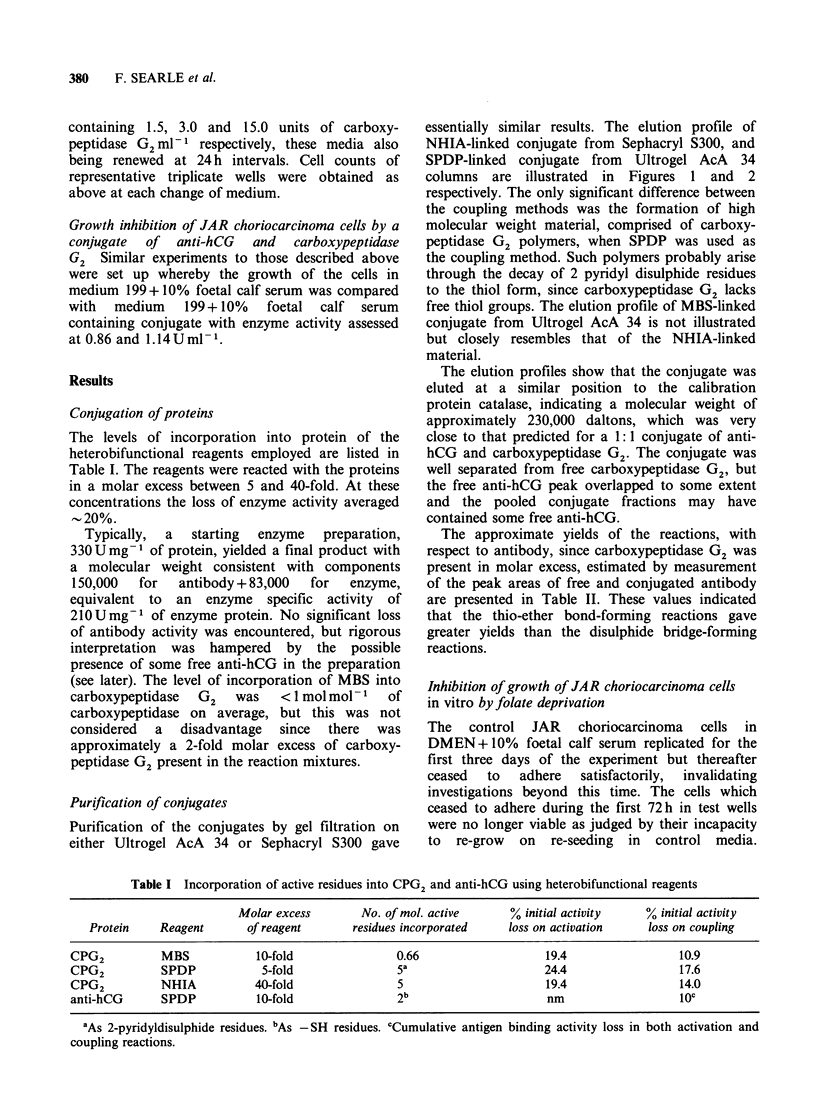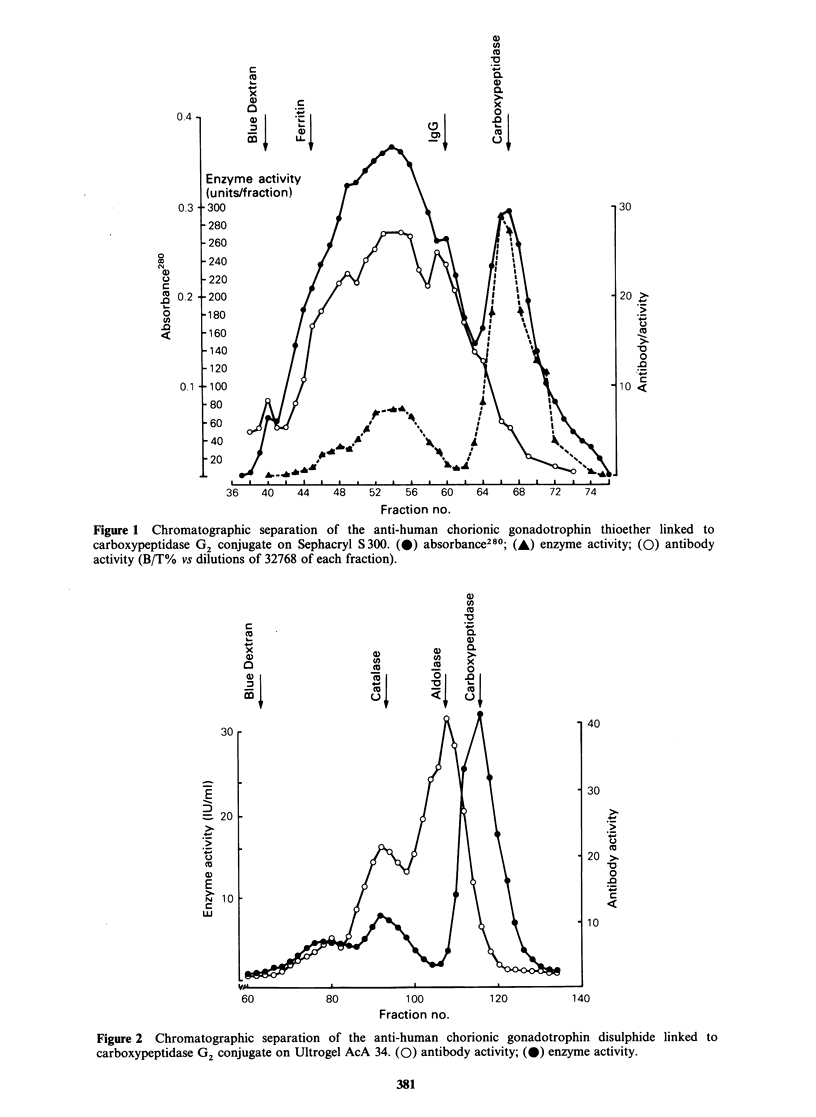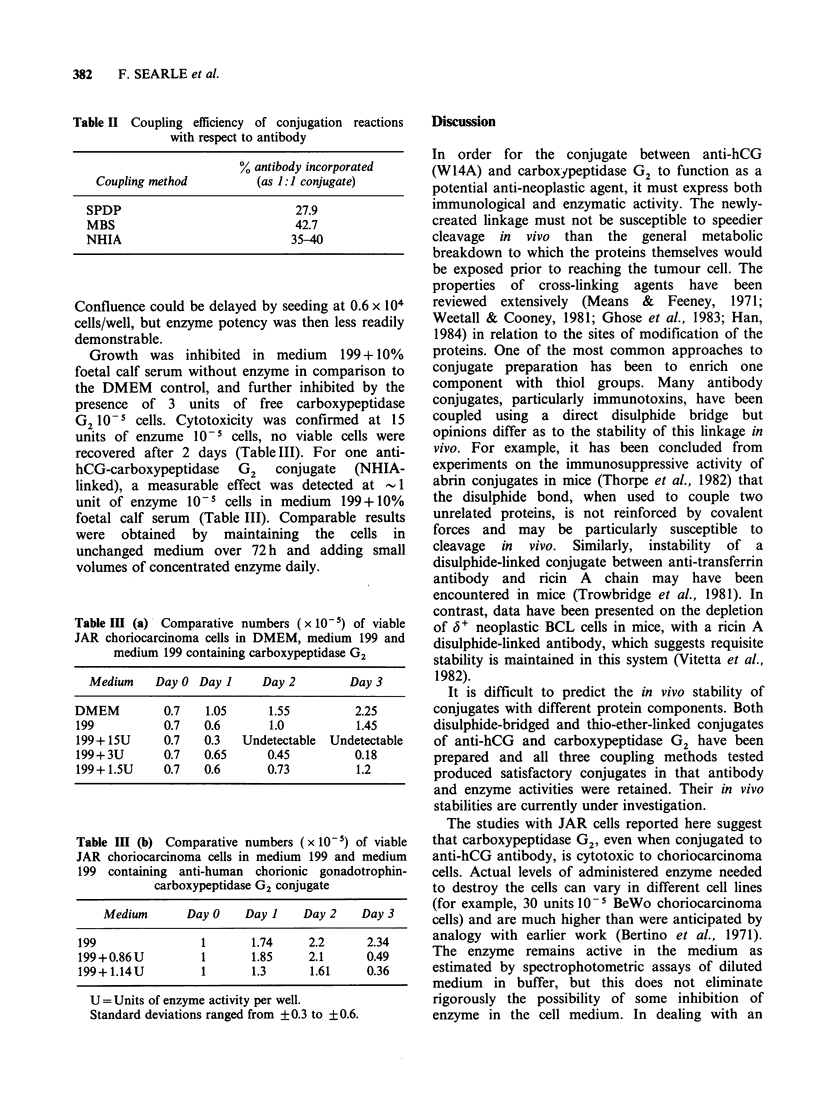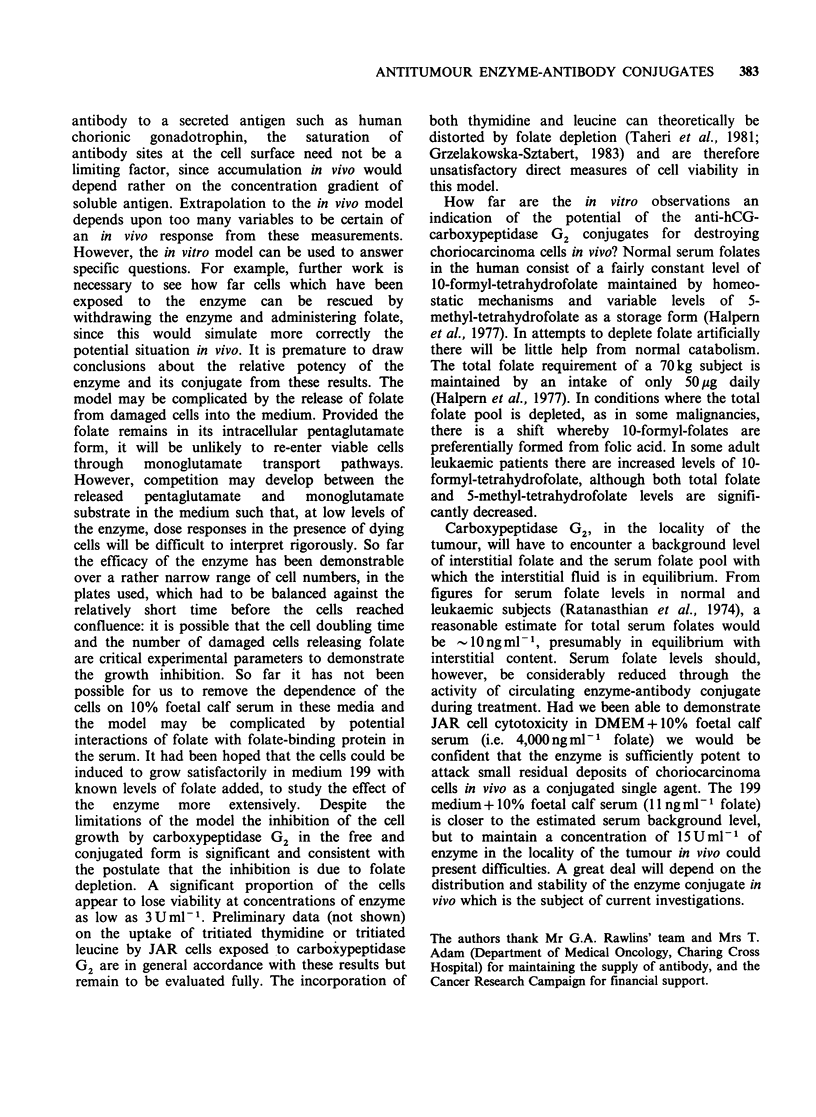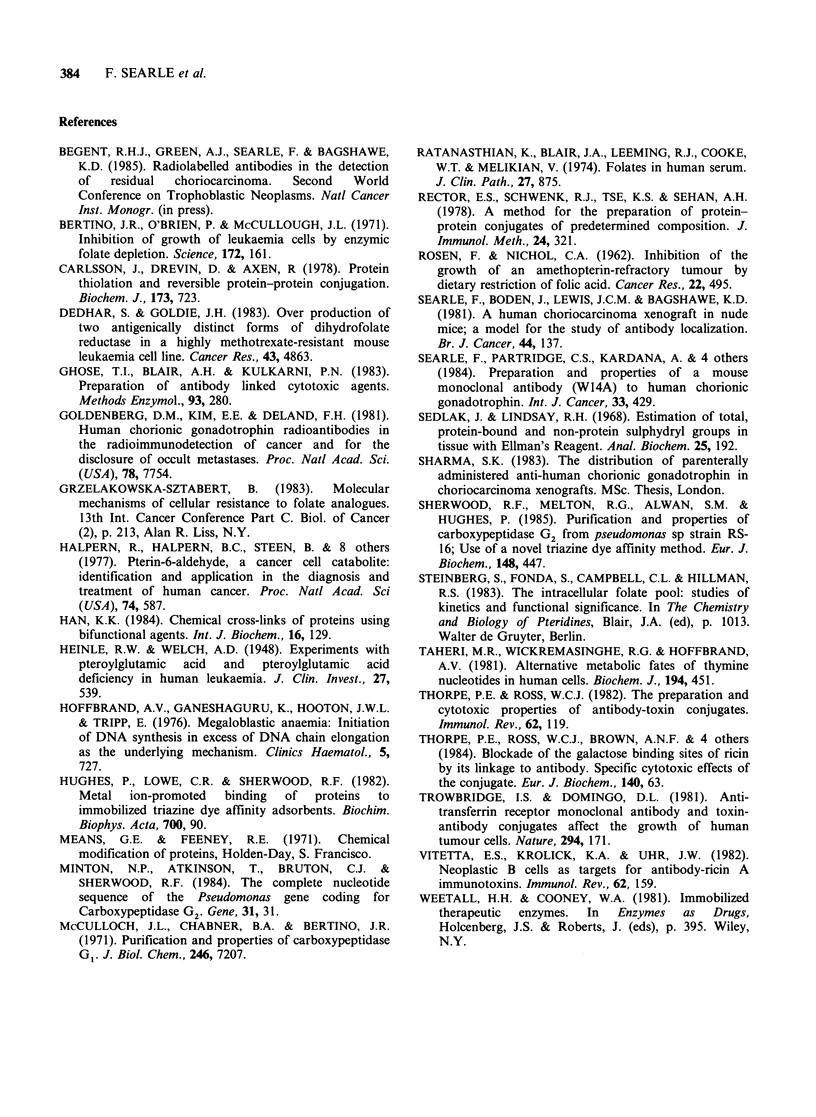# The potential of carboxypeptidase G2-antibody conjugates as anti-tumour agents. I. Preparation of antihuman chorionic gonadotrophin-carboxypeptidase G2 and cytotoxicity of the conjugate against JAR choriocarcinoma cells in vitro.

**DOI:** 10.1038/bjc.1986.62

**Published:** 1986-03

**Authors:** F. Searle, C. Bier, R. G. Buckley, S. Newman, R. B. Pedley, K. D. Bagshawe, R. G. Melton, S. M. Alwan, R. F. Sherwood

## Abstract

Carboxypeptidase G2, a zinc metalloenzyme isolated from Pseudomonas sp. strain RS-16, which catalyses the hydrolytic cleavage of reduced and non-reduced folates to pteroates and L-glutamate, has been linked to a monoclonal antibody (W14A) raised to human chorionic gonadotrophin. The coupling efficiency and retention of antibody and enzymatic activities are compared for three separate methods of preparing 1:1 conjugates. Preliminary in vitro studies on the cytotoxicity of the free enzyme and the conjugated enzyme towards JAR choriocarcinoma cells are reported. Despite the limitations of the in vitro model, it could be demonstrated that a significant proportion of 10(6) choriocarcinoma cells lost viability when exposed to either free or conjugated enzyme for 72 hours at concentrations of carboxypeptidase G2 of 1-3 units ml-1 of medium.


*Br. J. Cancer* (1986), **53**, 377–384

### The potential of carboxypeptidase $G_2$-antibody conjugates as anti-tumour agents. I. Preparation of antihuman chorionic gonadotrophin-carboxypeptidase $G_2$ and cytotoxicity of the conjugate against JAR choriocarcinoma cells *in vitro*

F. Searle[1], C. Bier[1], R.G. Buckley[1], S. Newman[1], R.B. Pedley[1], K.D. Bagshawe[1], R.G. Melton[2], S.M. Alwan[2] & R.F. Sherwood[2]

[1]*Cancer Research Campaign Laboratories, Charing Cross Hospital, Department of Medical Oncology, London W6 8RF;* [2]*Microbial Technology Laboratory, PHLS Centre for Applied Microbiology and Research, Porton Down, Salisbury, Wiltshire, UK.*

**Summary** Carboxypeptidase $G_2$, a zinc metalloenzyme isolated from *Pseudomonas sp.* strain RS-16, which catalyses the hydrolytic cleavage of reduced and non-reduced folates to pteroates and L-glutamate, has been linked to a monoclonal antibody (W14A) raised to human chorionic gonadotrophin. The coupling efficiency and retention of antibody and enzymatic activities are compared for three separate methods of preparing 1:1 conjugates. Preliminary *in vitro* studies on the cytotoxicity of the free enzyme and the conjugated enzyme towards JAR choriocarcinoma cells are reported. Despite the limitations of the *in vitro* model, it could be demonstrated that a significant proportion of $10^6$ choriocarcinoma cells lost viability when exposed to either free or conjugated enzyme for 72 hours at concentrations of carboxypeptidase $G_2$ of 1–3 units $ml^{-1}$ of medium.

Some chemotherapeutic anti-cancer agents function by entering cells and competing with metabolites used in DNA synthesis. Certain enzymes, with an inherently high degree of substrate specificity, may be capable of depriving a cell of essential metabolites and may fulfil this function from outside the cell. Selective delivery to malignant cells *in vivo*, by targeting the enzyme conjugated to an antibody which is directed at a tumour-associated antigen, at or near the cell surface, offers a potential means of achieving this.

Folate plays a central role in sustaining cell replication and cells depend upon a continued external supply. A major determinant of intracellular folate depletion can be dilution among cellular progeny. For example, DNA replication in Friend erythroleukaemia cells becomes compromised when the intracellular folate (ICF) falls to the range of $3 \times 10^5$ molecules per cell and the cell is incapable of further replication when the ICF pool is diminished to less than $1 \times 10^5$ molecules per cell (Steinberg *et al.*, 1983). The idea of depriving malignant cells of folate by diminishing extracellular folate is not new. A beneficial effect of dietary folate depletion in human leukaemia has been demonstrated (Heinle *et al.*, 1948), and the experimental Walker 256 adenocarcinoma of the rat is markedly inhibited by folate deficiency (Rosen *et al.*, 1962).

An enzyme, carboxypeptidase $G_1$, which catalyses the hydrolytic cleavage of reduced and non-reduced folates to pteroates and L-glutamate, was demonstrated to inhibit the growth of L1210 leukaemic cells, amongst others, *in vitro* (Bertino *et al.*, 1971). Both methotrexate-sensitive and methotrexate-resistant cell lines were similarly affected and this encouraged us to consider the choriocarcinoma model where methotrexate resistance may result from amplification of dihydrofolate reductase activity, as in a number of other malignancies (Dedhar *et al.*, 1983). Antibody-directed enzymatic deprivation of folate might be a valuable adjunct to conventional chemotherapy.

Carboxypeptidase $G_2$ (Sherwood *et al.*, 1985) a zinc metalloenzyme, isolated from *Pseudomonas sp.* strain RS-16, has very similar characteristics to carboxypeptidase $G_1$, and is a suitable candidate enzyme for linkage to antibodies against human chorionic gonadotrophin (hCG), expressed by syncytiotrophoblastic cells. Preliminary studies in CC3 choriocarcinoma xenografts in nude mice (Searle *et al.*, 1981) and radioimmunolocalisation studies in patients (Goldenberg *et al.*, 1981; Begent *et al.*, 1985) have already indicated that there is some selective retention of these antibodies in the locality of hCG-producing tumours. Autoradiography with iodine ($^{125}I$)-labelled antibodies suggests the major proportion remains extracellular

Correspondence: F. Searle.
Received 18 July 1985; and in revised form, 26 November 1985.

© The Macmillan Press Ltd., 1986

in the xenograft model (Sharma, 1983). The preparation of anti-hCG-carboxypeptidase $G_2$ conjugates has been undertaken to establish suitable methods to link the proteins without unacceptable losses of antibody or enzyme activity. Preliminary *in vitro* studies with JAR choriocarcinoma cells have been initiated in order to gain some insight into the requirements of local concentration, time of retention, and enzymatic activity required to produce a cytotoxic effect.

Three methods were used in this study to couple carboxypeptidase $G_2$ to anti-human chorionic gonadotrophin antibody. All were based on heterofunctional reagents which react with an amino group on one protein and a thiol residue on the second. A variety of coupling methods was chosen to enable the *in vivo* stability of the different bonds to be studied.

Carboxypeptidase $G_2$ does not possess any free thiol groups (Minton *et al.*, 1984) and was not considered suitable for the introduction of such residues because of loss of activity under the reaction conditions used for the reduction of 2-pyridyldisulphide groups. This limitation did not apply to the antibody, and hence it was this molecule which was thiolated. The reaction of the heterobifunctional reagents with amino groups occurs at approximately neutral pH and caused no major difficulties with the enzyme.

The heterobifunctional reagents used were N-succinimidyl-3-(2-pyridyldithio)-propionate (SPDP) which generates a disulphide bridge between the two protein molecules; the N-hydroxysuccinimide ester of iodoacetic acid (NHIA) which forms a thioether bond between the protein molecules and N-maleimidobenzoyl succinimide ester (MBS), the active maleimide bond of which forms a thioether bond with a thiol group on one protein, with the active ester acylating an amino group on the second protein.

### Materials and methods

### *Materials*

*Chemicals* N-Succinimidyl-3-(2-pyridyldithio) propionate (SPDP) was purchased from Pharmacia Ltd., Milton Keynes, UK; N-hydroxysuccinimide and N,N-dicyclohexylcarbodiimide were purchased from Aldrich Ltd., Gillingham, Dorset, UK; N-maleimidobenzoyl succinimide ester (MBS) and 5,5′-dithiobis-(2-nitrobenzoic acid) (DTNB) were purchased from Sigma Ltd, Poole, Dorset, UK; 1,4-dioxan (which was applied to a column of aluminium oxide before use), iodoacetic acid and all other chemicals, which were of 'Analar' grade, were purchased from B.D.H. Ltd., Poole, Dorset, UK.

*Biological materials* Carboxypeptidase $G_2$ ($330\,U\,mg^{-1}$) was produced by the Microbial Technology Laboratory following a previously described protocol (Sherwood *et al.*, 1985). Antihuman chorionic gonadotrophin, anti-hCG W14A, an $IgG_1$ mouse monoclonal antibody was prepared from ascites as reported previously (Searle *et al.*, 1984). JAR choriocarcinoma cells were a gift from Professor R. Pattillo.

*Buffers* 0.05 M sodium phosphate buffer, pH 7.5 containing 0.15 M NaCl; 0.1 M sodium acetate, pH 7.0, containing 0.15 M NaCl; 0.1 M Tris-HCl, pH 7.3, containing 0.2 mM $ZnCl_2$; phosphate-buffered saline (PBS): NaCl 0.8%, w/v, KCl 0.02%, w/v, $Na_2HPO_4$ 0.13%, w/v, $KH_2PO_4$ 0.02%, w/v; pH 7.4.

*Chromatographic materials* Bio-Gel P30 was purchased from Bio-Rad Ltd, Watford, Herts, UK, Sephacryl S300 Superfine and Sephadex G25M prepacked (PD 10) columns were purchased from Pharmacia Ltd, Milton Keynes, UK, and Ultrogel AcA 34 was purchased from LKB Ltd, Croydon, UK.

*Media for cell culture* DMEM with 20 mM HEPES buffer, supplemented with 10% foetal calf serum (Flow Laboratories), penicillin $100\,U\,ml^{-1}$ (Glaxo), streptomycin sulphate $100\,\mu g\,ml^{-1}$, (Evans Medical Ltd, Middlesex) and L-glutamine 2 mM (Gibco). DMEM contains $4.0\,mg\,l^{-1}$ of folate. Folate-depleted medium, was Medium 199 with Earle's salts supplemented as above and, contains $0.01\,mg\,l^{-1}$ of folate. The folate content of the foetal calf serum batch was $9.3\,\mu g\,l^{-1}$.

### *Methods*

*Determination of biological activities* Carboxypeptidase $G_2$ activity was measured spectrophotometrically at 37°C in tris-HCl buffer with 0.06 mM methotrexate as substrate in a total volume of 1 ml. The reaction was started by the addition of enzyme and followed by the decrease in absorbance at 320 nm (Hughes *et al.*, 1982; McCullough *et al.*, 1971).

Anti-hCG activity relative to starting material was measured as described previously (Searle *et al.*, 1984). Antiserum dilution curves were set up as follows: each tube contained a mixture of phosphate buffer (200 µl), antibody or conjugate at different dilutions in 1/400 normal mouse serum in phosphate buffer (50 µl), $I^{125}$-hCG (50 µl of stock solution specific activity $180\,\mu Ci\,\mu g^{-1}$ diluted to achieve 60–70,000 c.p.m.). Reaction mixtures were incubated for 16 h at room temperature and then rabbit antimouse immunoglobulin (50 $\mu$l) added to a final dilution of 1/280 together with 10% polyethylene glycol 6000 (50 $\mu$l). The whole was incubated for a further 2 h at room temperature and then filtered and counted (Searle *et al.*, 1984).

*Thiolation of anti-hCG* One hundred $\mu$l of a stock solution of SPDP reagent (2.08 mg ml$^{-1}$) in ethanol were added, with stirring, to a solution of anti-hCG (10 mg) in phosphate buffer (3.5 ml). After stirring at room temperature for 30 min, the mixture was applied to a column of BioGel P30 (15 mm x 240 mm) pre-equilibrated with acetate buffer and eluted with the same buffer. Immunoglobulin-containing fractions were tested (Carlsson *et al.*, 1978) to determine the degree of incorporation of 2-pyridyldisulphide residues.

Dithiothreitol (3.9 mg) was added to the substituted immunoglobulin solution (5.1 ml). After stirring for 20 min at room temperature, the mixture was applied to a column of BioGel P30 (15 mm x 240 mm) pre-equilibrated with phosphate buffer, and eluted (1.75 ml fractions) with the same buffer. Fractions containing anti-hCG (7 ml) were pooled.

*Preparation of anti-hCG-carboxypeptidase $G_2$ conjugate using NHIA* One hundred and twenty-three $\mu$l of a stock solution of NHIA (12.5 mg ml$^{-1}$) (Rector *et al.*, 1978) in dimethylformamide were added with stirring, in portions, to a solution of carboxypeptidase $G_2$ (11 mg) in phosphate buffer (2.5 ml). After stirring for 40 min at room temperature, the mixture was applied to a column of Bio-Rad P30 (15 mm x 240 mm), pre-equilibrated with phosphate buffer, and eluted (1.75 ml fractions) with the same buffer. The carboxypeptidase $G_2$ fraction (7 ml) was tested spectrophotometrically to confirm the introduction of iodoacetyl groups (Thorpe *et al.*, 1984).

The iodoacetylated carboxypeptidase $G_2$ was mixed with thiolated anti-hCG prepared as above, and the mixture concentrated to 2.5 ml by ultrafiltration and allowed to react for 60 h at room temperature. Anti-hCG-carboxypeptidase $G_2$ conjugate was purified by column chromatography on Sephacryl S300 Superfine (26 mm x 650 mm), pre-equilibrated and eluted with phosphate buffer. Fractions (2.75 ml) containing antibody and enzyme activity were pooled from several preparations to give a total of 160 ml, concentrated 10-fold by ultrafiltration and stored at $-70$°C.

*Preparation of anti-hCG-carboxypeptidase $G_2$ comjugate using SPDP* Fifty $\mu$l of a stock solution of SPDP reagent (2.1 mg ml$^{-1}$) in ethanol were added with stirring, to a solution of carboxypeptidase $G_2$ (5.5 mg) in phosphate buffer (2.4 ml). After stirring for 40 min at room temperature, the mixture was applied to a Sephadex G25M (PD10) column, pre-equilibrated and eluted with phosphate buffer. An aliquot was retained for the determination of 2-pyridyldisulphide residue-incorporation as before. Fractions (0.5 ml) were collected and assayed for enzyme activity. The peak fractions (3.5 ml) were mixed with thiolated anti-hCG (5 mg, 3.5 ml), the mixture concentrated to 2.5 ml by ultrafiltration, and allowed to react for 60 h at 4°C. Anti-hCG-carboxypeptidase $G_2$ conjugate was purified by chromatography on a column of Ultrogel AcA34 (22 mm x 900 mm), pre-equilibrated and eluted with phosphate buffer. Fractions (2 ml) were collected and assayed as before.

*Preparation of anti-hCG-carboxypeptidase $G_2$ conjugate using MBS* Fifty $\mu$l of a stock solution of MBS (4.2 mg ml$^{-1}$ in tetrahydrofuran) were added with stirring to a solution of carboxypeptidase $G_2$ (5.5 mg) in phosphate buffer (2.4 ml). After stirring for 40 min, the mixture was applied to a Sephadex G25M (PD10) column pre-equilibrated and eluted with phosphate buffer. Fractions (0.5 ml) were collected and assayed for enzyme activity. Peak fractions (3.5 ml) were pooled and an aliquot retained for the determination of MBS incorporation by measuring the decrease of the thiol content on its addition to mercaptoethanol (100 nmol) using Ellman's reagent (DTNB) (Sedlack & Lindsay, 1968). The pooled, activated enzyme was mixed with thiolated anti-hCG, (5 mg, 3.5 ml) the mixture concentrated to 2.5 ml by ultrafiltration and allowed to react for 60 h at 4°C. Anti-hCG-carboxypeptidase $G_2$ was isolated by chromatography on a column of Ultrogel AcA 34 (22 mm x 900 mm) pre-equilibrated and eluted with PBS. Fractions (2 ml) were collected and assayed as before.

*Growth inhibition of JAR choriocarcinoma cells by carboxypeptidase $G_2$* Cells were trypsinized (0.5% trypsin and 0.02% EDTA in saline) from stock flasks and seeded at $1 \times 10^5$ cells/well in 1 ml DMEM + 10% foetal calf serum per well for controls and 1 ml 199 + 10% foetal calf serum for folate-depleted controls. After 24 h, viable cells in triplicate wells were estimated by trypsinization of the adherent cells and counting with a haemocytometer using trypan blue uptake. The stock media were renewed at 24 h intervals in further control wells for 4 days, while experimental triplicate wells were exposed to medium 199

containing 1.5, 3.0 and 15.0 units of carboxypeptidase $G_2\,ml^{-1}$ respectively, these media also being renewed at 24 h intervals. Cell counts of representative triplicate wells were obtained as above at each change of medium.

*Growth inhibition of JAR choriocarcinoma cells by a conjugate of anti-hCG and carboxypeptidase $G_2$* Similar experiments to those described above were set up whereby the growth of the cells in medium 199+10% foetal calf serum was compared with medium 199+10% foetal calf serum containing conjugate with enzyme activity assessed at 0.86 and $1.14\,U\,ml^{-1}$.

### Results

### *Conjugation of proteins*

The levels of incorporation into protein of the heterobifunctional reagents employed are listed in Table I. The reagents were reacted with the proteins in a molar excess between 5 and 40-fold. At these concentrations the loss of enzyme activity averaged ~20%.

Typically, a starting enzyme preparation, $330\,U\,mg^{-1}$ of protein, yielded a final product with a molecular weight consistent with components 150,000 for antibody+83,000 for enzyme, equivalent to an enzyme specific activity of $210\,U\,mg^{-1}$ of enzyme protein. No significant loss of antibody activity was encountered, but rigorous interpretation was hampered by the possible presence of some free anti-hCG in the preparation (see later). The level of incorporation of MBS into carboxypeptidase $G_2$ was $<1\,mol\,mol^{-1}$ of carboxypeptidase on average, but this was not considered a disadvantage since there was approximately a 2-fold molar excess of carboxypeptidase $G_2$ present in the reaction mixtures.

### *Purification of conjugates*

Purification of the conjugates by gel filtration on either Ultrogel AcA 34 or Sephacryl S300 gave essentially similar results. The elution profile of NHIA-linked conjugate from Sephacryl S300, and SPDP-linked conjugate from Ultrogel AcA 34 columns are illustrated in Figures 1 and 2 respectively. The only significant difference between the coupling methods was the formation of high molecular weight material, comprised of carboxypeptidase $G_2$ polymers, when SPDP was used as the coupling method. Such polymers probably arise through the decay of 2 pyridyl disulphide residues to the thiol form, since carboxypeptidase $G_2$ lacks free thiol groups. The elution profile of MBS-linked conjugate from Ultrogel AcA 34 is not illustrated but closely resembles that of the NHIA-linked material.

The elution profiles show that the conjugate was eluted at a similar position to the calibration protein catalase, indicating a molecular weight of approximately 230,000 daltons, which was very close to that predicted for a 1:1 conjugate of anti-hCG and carboxypeptidase $G_2$. The conjugate was well separated from free carboxypeptidase $G_2$, but the free anti-hCG peak overlapped to some extent and the pooled conjugate fractions may have contained some free anti-hCG.

The approximate yields of the reactions, with respect to antibody, since carboxypeptidase $G_2$ was present in molar excess, estimated by measurement of the peak areas of free and conjugated antibody are presented in Table II. These values indicated that the thio-ether bond-forming reactions gave greater yields than the disulphide bridge-forming reactions.

### *Inhibition of growth of JAR choriocarcinoma cells* in vitro *by folate deprivation*

The control JAR choriocarcinoma cells in DMEN+10% foetal calf serum replicated for the first three days of the experiment but thereafter ceased to adhere satisfactorily, invalidating investigations beyond this time. The cells which ceased to adhere during the first 72 h in test wells were no longer viable as judged by their incapacity to re-grow on re-seeding in control media.

**Table I** Incorporation of active residues into $CPG_2$ and anti-hCG using heterobifunctional reagents

| *Protein* | *Reagent* | *Molar excess of reagent* | *No. of mol. active residues incorporated* | *% initial activity loss on activation* | *% initial activity loss on coupling* |
|---|---|---|---|---|---|
| $CPG_2$ | MBS | 10-fold | 0.66 | 19.4 | 10.9 |
| $CPG_2$ | SPDP | 5-fold | 5[a] | 24.4 | 17.6 |
| $CPG_2$ | NHIA | 40-fold | 5 | 19.4 | 14.0 |
| anti-hCG | SPDP | 10-fold | 2[b] | nm | 10[c] |

[a]As 2-pyridyldisulphide residues. [b]As −SH residues. [c]Cumulative antigen binding activity loss in both activation and coupling reactions.

**Figure 1** Chromatographic separation of the anti-human chorionic gonadotrophin thioether linked to carboxypeptidase $G_2$ conjugate on Sephacryl S300. (●) absorbance280; (▲) enzyme activity; (○) antibody activity (B/T% *vs* dilutions of 32768 of each fraction).

**Figure 2** Chromatographic separation of the anti-human chorionic gonadotrophin disulphide linked to carboxypeptidase $G_2$ conjugate on Ultrogel AcA 34. (○) antibody activity; (●) enzyme activity.

**Table II** Coupling efficiency of conjugation reactions with respect to antibody

| *Coupling method* | *% antibody incorporated (as 1:1 conjugate)* |
|---|---|
| SPDP | 27.9 |
| MBS | 42.7 |
| NHIA | 35–40 |

Confluence could be delayed by seeding at $0.6 \times 10^4$ cells/well, but enzyme potency was then less readily demonstrable.

Growth was inhibited in medium 199+10% foetal calf serum without enzyme in comparison to the DMEM control, and further inhibited by the presence of 3 units of free carboxypeptidase $G_2$ $10^{-5}$ cells. Cytotoxicity was confirmed at 15 units of enzume $10^{-5}$ cells, no viable cells were recovered after 2 days (Table III). For one anti-hCG-carboxypeptidase $G_2$ conjugate (NHIA-linked), a measurable effect was detected at ~1 unit of enzyme $10^{-5}$ cells in medium 199+10% foetal calf serum (Table III). Comparable results were obtained by maintaining the cells in unchanged medium over 72h and adding small volumes of concentrated enzyme daily.

**Table III (a)** Comparative numbers ($\times 10^{-5}$) of viable JAR choriocarcinoma cells in DMEM, medium 199 and medium 199 containing carboxypeptidase $G_2$

| *Medium* | *Day 0* | *Day 1* | *Day 2* | *Day 3* |
|---|---|---|---|---|
| DMEM | 0.7 | 1.05 | 1.55 | 2.25 |
| 199 | 0.7 | 0.6 | 1.0 | 1.45 |
| 199+15U | 0.7 | 0.3 | Undetectable | Undetectable |
| 199+3U | 0.7 | 0.65 | 0.45 | 0.18 |
| 199+1.5U | 0.7 | 0.6 | 0.73 | 1.2 |

**Table III (b)** Comparative numbers ($\times 10^{-5}$) of viable JAR choriocarcinoma cells in medium 199 and medium 199 containing anti-human chorionic gonadotrophin-carboxypeptidase $G_2$ conjugate

| *Medium* | *Day 0* | *Day 1* | *Day 2* | *Day 3* |
|---|---|---|---|---|
| 199 | 1 | 1.74 | 2.2 | 2.34 |
| 199+0.86U | 1 | 1.85 | 2.1 | 0.49 |
| 199+1.14U | 1 | 1.3 | 1.61 | 0.36 |

U = Units of enzyme activity per well.
Standard deviations ranged from ±0.3 to ±0.6.

### Discussion

In order for the conjugate between anti-hCG (W14A) and carboxypeptidase $G_2$ to function as a potential anti-neoplastic agent, it must express both immunological and enzymatic activity. The newly-created linkage must not be susceptible to speedier cleavage *in vivo* than the general metabolic breakdown to which the proteins themselves would be exposed prior to reaching the tumour cell. The properties of cross-linking agents have been reviewed extensively (Means & Feeney, 1971; Weetall & Cooney, 1981; Ghose *et al.*, 1983; Han, 1984) in relation to the sites of modification of the proteins. One of the most common approaches to conjugate preparation has been to enrich one component with thiol groups. Many antibody conjugates, particularly immunotoxins, have been coupled using a direct disulphide bridge but opinions differ as to the stability of this linkage *in vivo*. For example, it has been concluded from experiments on the immunosuppressive activity of abrin conjugates in mice (Thorpe *et al.*, 1982) that the disulphide bond, when used to couple two unrelated proteins, is not reinforced by covalent forces and may be particularly susceptible to cleavage *in vivo*. Similarly, instability of a disulphide-linked conjugate between anti-transferrin antibody and ricin A chain may have been encountered in mice (Trowbridge *et al.*, 1981). In contrast, data have been presented on the depletion of $\delta^+$ neoplastic BCL cells in mice, with a ricin A disulphide-linked antibody, which suggests requisite stability is maintained in this system (Vitetta *et al.*, 1982).

It is difficult to predict the *in vivo* stability of conjugates with different protein components. Both disulphide-bridged and thio-ether-linked conjugates of anti-hCG and carboxypeptidase $G_2$ have been prepared and all three coupling methods tested produced satisfactory conjugates in that antibody and enzyme activities were retained. Their *in vivo* stabilities are currently under investigation.

The studies with JAR cells reported here suggest that carboxypeptidase $G_2$, even when conjugated to anti-hCG antibody, is cytotoxic to choriocarcinoma cells. Actual levels of administered enzyme needed to destroy the cells can vary in different cell lines (for example, 30 units $10^{-5}$ BeWo choriocarcinoma cells) and are much higher than were anticipated by analogy with earlier work (Bertino *et al.*, 1971). The enzyme remains active in the medium as estimated by spectrophotometric assays of diluted medium in buffer, but this does not eliminate rigorously the possibility of some inhibition of enzyme in the cell medium. In dealing with an antibody to a secreted antigen such as human chorionic gonadotrophin, the saturation of antibody sites at the cell surface need not be a limiting factor, since accumulation *in vivo* would depend rather on the concentration gradient of soluble antigen. Extrapolation to the *in vivo* model depends upon too many variables to be certain of an *in vivo* response from these measurements. However, the *in vitro* model can be used to answer specific questions. For example, further work is necessary to see how far cells which have been exposed to the enzyme can be rescued by withdrawing the enzyme and administering folate, since this would simulate more correctly the potential situation *in vivo*. It is premature to draw conclusions about the relative potency of the enzyme and its conjugate from these results. The model may be complicated by the release of folate from damaged cells into the medium. Provided the folate remains in its intracellular pentaglutamate form, it will be unlikely to re-enter viable cells through monoglutamate transport pathways. However, competition may develop between the released pentaglutamate and monoglutamate substrate in the medium such that, at low levels of the enzyme, dose responses in the presence of dying cells will be difficult to interpret rigorously. So far the efficacy of the enzyme has been demonstrable over a rather narrow range of cell numbers, in the plates used, which had to be balanced against the relatively short time before the cells reached confluence: it is possible that the cell doubling time and the number of damaged cells releasing folate are critical experimental parameters to demonstrate the growth inhibition. So far it has not been possible for us to remove the dependence of the cells on 10% foetal calf serum in these media and the model may be complicated by potential interactions of folate with folate-binding protein in the serum. It had been hoped that the cells could be induced to grow satisfactorily in medium 199 with known levels of folate added, to study the effect of the enzyme more extensively. Despite the limitations of the model the inhibition of the cell growth by carboxypeptidase $G_2$ in the free and conjugated form is significant and consistent with the postulate that the inhibition is due to folate depletion. A significant proportion of the cells appear to lose viability at concentrations of enzyme as low as $3\,U\,ml^{-1}$. Preliminary data (not shown) on the uptake of tritiated thymidine or tritiated leucine by JAR cells exposed to carboxypeptidase $G_2$ are in general accordance with these results but remain to be evaluated fully. The incorporation of both thymidine and leucine can theoretically be distorted by folate depletion (Taheri *et al.*, 1981; Grzelakowska-Sztabert, 1983) and are therefore unsatisfactory direct measures of cell viability in this model.

How far are the *in vitro* observations an indication of the potential of the anti-hCG-carboxypeptidase $G_2$ conjugates for destroying choriocarcinoma cells *in vivo*? Normal serum folates in the human consist of a fairly constant level of 10-formyl-tetrahydrofolate maintained by homeostatic mechanisms and variable levels of 5-methyl-tetrahydrofolate as a storage form (Halpern *et al.*, 1977). In attempts to deplete folate artificially there will be little help from normal catabolism. The total folate requirement of a 70 kg subject is maintained by an intake of only $50\,\mu g$ daily (Halpern *et al.*, 1977). In conditions where the total folate pool is depleted, as in some malignancies, there is a shift whereby 10-formyl-folates are preferentially formed from folic acid. In some adult leukaemic patients there are increased levels of 10-formyl-tetrahydrofolate, although both total folate and 5-methyl-tetrahydrofolate levels are significantly decreased.

Carboxypeptidase $G_2$, in the locality of the tumour, will have to encounter a background level of interstitial folate and the serum folate pool with which the interstitial fluid is in equilibrium. From figures for serum folate levels in normal and leukaemic subjects (Ratanasthian *et al.*, 1974), a reasonable estimate for total serum folates would be $\sim 10\,ng\,ml^{-1}$, presumably in equilibrium with interstitial content. Serum folate levels should, however, be considerably reduced through the activity of circulating enzyme-antibody conjugate during treatment. Had we been able to demonstrate JAR cell cytotoxicity in DMEM + 10% foetal calf serum (i.e. $4{,}000\,ng\,ml^{-1}$ folate) we would be confident that the enzyme is sufficiently potent to attack small residual deposits of choriocarcinoma cells *in vivo* as a conjugated single agent. The 199 medium + 10% foetal calf serum ($11\,ng\,ml^{-1}$ folate) is closer to the estimated serum background level, but to maintain a concentration of $15\,U\,ml^{-1}$ of enzyme in the locality of the tumour *in vivo* could present difficulties. A great deal will depend on the distribution and stability of the enzyme conjugate *in vivo* which is the subject of current investigations.

The authors thank Mr G.A. Rawlins' team and Mrs T. Adam (Department of Medical Oncology, Charing Cross Hospital) for maintaining the supply of antibody, and the Cancer Research Campaign for financial support.

### References

BEGENT, R.H.J., GREEN, A.J., SEARLE, F. & BAGSHAWE, K.D. (1985). Radiolabelled antibodies in the detection of residual choriocarcinoma. Second World Conference on Trophoblastic Neoplasms. *Natl Cancer Inst. Monogr.* (in press).

BERTINO, J.R., O'BRIEN, P. & McCULLOUGH, J.L. (1971). Inhibition of growth of leukaemia cells by enzymic folate depletion. *Science*, **172**, 161.

CARLSSON, J., DREVIN, D. & AXEN, R (1978). Protein thiolation and reversible protein–protein conjugation. *Biochem. J.*, **173**, 723.

DEDHAR, S. & GOLDIE, J.H. (1983). Over production of two antigenically distinct forms of dihydrofolate reductase in a highly methotrexate-resistant mouse leukaemia cell line. *Cancer Res.*, **43**, 4863.

GHOSE, T.I., BLAIR, A.H. & KULKARNI, P.N. (1983). Preparation of antibody linked cytotoxic agents. *Methods Enzymol.*, **93**, 280.

GOLDENBERG, D.M., KIM, E.E. & DELAND, F.H. (1981). Human chorionic gonadotrophin radioantibodies in the radioimmunodetection of cancer and for the disclosure of occult metastases. *Proc. Natl Acad. Sci. (USA)*, **78**, 7754.

GRZELAKOWSKA-SZTABERT, B. (1983). Molecular mechanisms of cellular resistance to folate analogues. 13th Int. Cancer Conference Part C. Biol. of Cancer (2), p. 213, Alan R. Liss, N.Y.

HALPERN, R., HALPERN, B.C., STEEN, B. & 8 others (1977). Pterin-6-aldehyde, a cancer cell catabolite: identification and application in the diagnosis and treatment of human cancer. *Proc. Natl Acad. Sci (USA)*, **74**, 587.

HAN, K.K. (1984). Chemical cross-links of proteins using bifunctional agents. *Int. J. Biochem.*, **16**, 129.

HEINLE, R.W. & WELCH, A.D. (1948). Experiments with pteroylglutamic acid and pteroylglutamic acid deficiency in human leukaemia. *J. Clin. Invest.*, **27**, 539.

HOFFBRAND, A.V., GANESHAGURU, K., HOOTON, J.W.L. & TRIPP, E. (1976). Megaloblastic anaemia: Initiation of DNA synthesis in excess of DNA chain elongation as the underlying mechanism. *Clinics Haematol.*, **5**, 727.

HUGHES, P., LOWE, C.R. & SHERWOOD, R.F. (1982). Metal ion-promoted binding of proteins to immobilized triazine dye affinity adsorbents. *Biochim. Biophys. Acta*, **700**, 90.

MEANS, G.E. & FEENEY, R.E. (1971). Chemical modification of proteins, Holden-Day, S. Francisco.

MINTON, N.P., ATKINSON, T., BRUTON, C.J. & SHERWOOD, R.F. (1984). The complete nucleotide sequence of the *Pseudomonas* gene coding for Carboxypeptidase $G_2$. *Gene*, **31**, 31.

McCULLOCH, J.L., CHABNER, B.A. & BERTINO, J.R. (1971). Purification and properties of carboxypeptidase $G_1$. *J. Biol. Chem.*, **246**, 7207.

RATANASTHIAN, K., BLAIR, J.A., LEEMING, R.J., COOKE, W.T. & MELIKIAN, V. (1974). Folates in human serum. *J. Clin. Path.*, **27**, 875.

RECTOR, E.S., SCHWENK, R.J., TSE, K.S. & SEHAN, A.H. (1978). A method for the preparation of protein–protein conjugates of predetermined composition. *J. Immunol. Meth.*, **24**, 321.

ROSEN, F. & NICHOL, C.A. (1962). Inhibition of the growth of an amethopterin-refractory tumour by dietary restriction of folic acid. *Cancer Res.*, **22**, 495.

SEARLE, F., BODEN, J., LEWIS, J.C.M. & BAGSHAWE, K.D. (1981). A human choriocarcinoma xenograft in nude mice; a model for the study of antibody localization. *Br. J. Cancer*, **44**, 137.

SEARLE, F., PARTRIDGE, C.S., KARDANA, A. & 4 others (1984). Preparation and properties of a mouse monoclonal antibody (W14A) to human chorionic gonadotrophin. *Int. J. Cancer*, **33**, 429.

SEDLAK, J. & LINDSAY, R.H. (1968). Estimation of total, protein-bound and non-protein sulphydryl groups in tissue with Ellman's Reagent. *Anal. Biochem.* **25**, 192.

SHARMA, S.K. (1983). The distribution of parenterally administered anti-human chorionic gonadotrophin in choriocarcinoma xenografts. MSc. Thesis, London.

SHERWOOD, R.F., MELTON, R.G., ALWAN, S.M. & HUGHES, P. (1985). Purification and properties of carboxypeptidase $G_2$ from *pseudomonas* sp strain RS-16; Use of a novel triazine dye affinity method. *Eur. J. Biochem.*, **148**, 447.

STEINBERG, S., FONDA, S., CAMPBELL, C.L. & HILLMAN, R.S. (1983). The intracellular folate pool: studies of kinetics and functional significance. In *The Chemistry and Biology of Pteridines*, Blair, J.A. (ed), p. 1013. Walter de Gruyter, Berlin.

TAHERI, M.R., WICKREMASINGHE, R.G. & HOFFBRAND, A.V. (1981). Alternative metabolic fates of thymine nucleotides in human cells. *Biochem. J.*, **194**, 451.

THORPE, P.E. & ROSS, W.C.J. (1982). The preparation and cytotoxic properties of antibody-toxin conjugates. *Immunol. Rev.*, **62**, 119.

THORPE, P.E., ROSS, W.C.J., BROWN, A.N.F. & 4 others (1984). Blockade of the galactose binding sites of ricin by its linkage to antibody. Specific cytotoxic effects of the conjugate. *Eur. J. Biochem.*, **140**, 63.

TROWBRIDGE, I.S. & DOMINGO, D.L. (1981). Anti-transferrin receptor monoclonal antibody and toxin-antibody conjugates affect the growth of human tumour cells. *Nature*, **294**, 171.

VITETTA, E.S., KROLICK, K.A. & UHR, J.W. (1982). Neoplastic B cells as targets for antibody-ricin A immunotoxins. *Immunol. Rev.*, **62**, 159.

WEETALL, H.H. & COONEY, W.A. (1981). Immobilized therapeutic enzymes. In *Enzymes as Drugs*, Holcenberg, J.S. & Roberts, J. (eds), p. 395. Wiley, N.Y.